# Supplementary material for: Elevated Ozone Reduces the Quality of Tea Leaves but May Improve the Resistance of Tea Plants
Source: Plants (Basel). 2024 Apr 16;13(8):1108. doi: 10.3390/plants13081108 (PMC11054534; doi:10.3390/plants13081108)
Supplement: Supplementary file 1 [file plants-13-01108-s001.zip › plants-2901350-supplementary.pdf]

Table S1 The retention time of caffeine and individual catechins.

Compounds	CAF	C	EC	EGC	ECG	EGCG
Retention time (min)	21.253	18.335	25.323	16.388	37.403	26.005

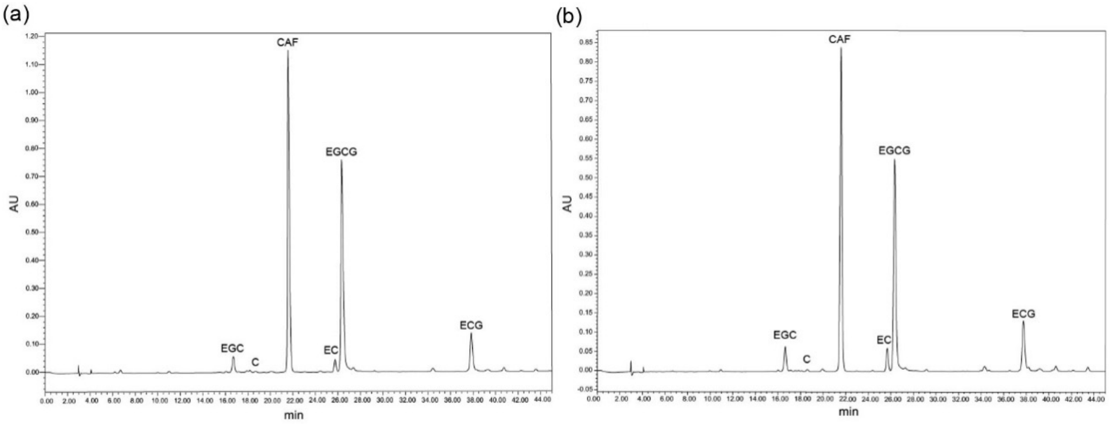

Figure S1. HPLC chromatograms of tea leaves under (a) ambient air and (b) elevated O_3 treatment. C, catechin; EC, epicatechin; ECG, epicatechin gallate; EGC, epigallocatechin; EGCG, epigallocatechin gallate; CAF, caffeine.

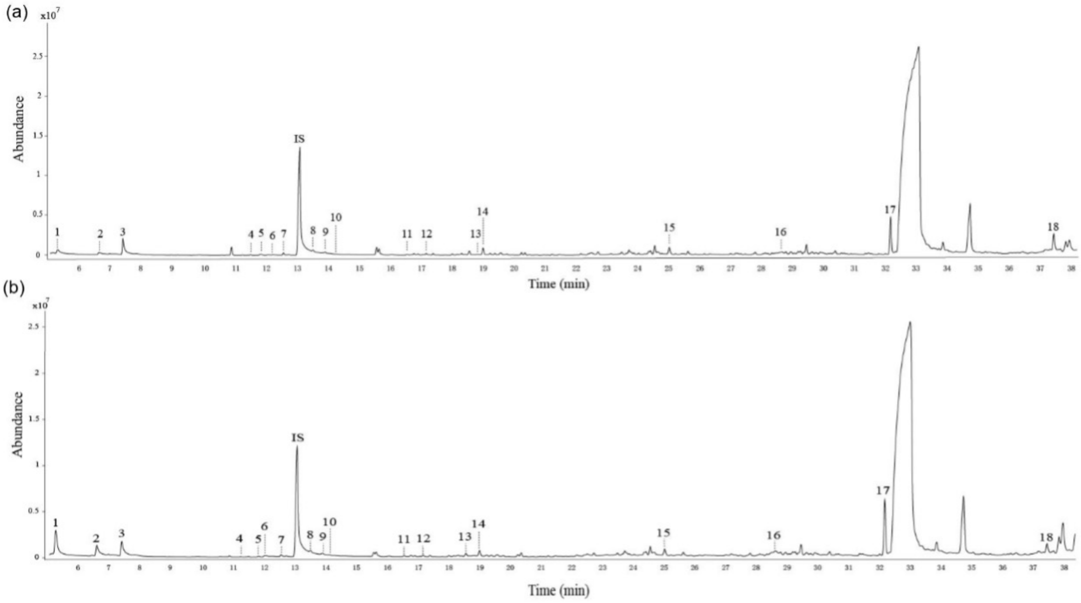

Figure S2. The total ion chromatogram of volatile components in tea leaves under (a) ambient air and (b) elevated O_3 treatment. 1, Toluene; 2, E-2-hexenal; 3, Cyclohexanol; 4, Undecane; 5, 2-Ethyl-1-hexanol; 6, Benzyl alcohol; 7, Dodecane; 8, Trans-Furanic linalool oxid; 9, 2,4-Dimethyl-1-heptanol; 10, Hexyl octyl ether; 11, 1-Methoxyadamantane; 12, Tridecane; 13, Geraniol; 14, Pentadecane; 15, 2,4-Di-tert-butylphenol; 16, 6-epi-shyobunol; 17, Neophytadiene; 18, Phytol; IS, n-octanol.
